# Fetal Alcohol Spectrum Disorder: The Caring and Financial Burden to Caregivers—A Scoping Review

**DOI:** 10.1111/dar.14071

**Published:** 2025-05-04

**Authors:** Josie Tait, Anita Gibbs, Jessica McCormack, Holly Wilson, Joanna Ting Wai Chu

**Affiliations:** ^1^ Social and Community Health School of Population Health, The University of Auckland Auckland New Zealand; ^2^ Sociology, Gender Studies and Criminology University of Otago Dunedin New Zealand; ^3^ Department of Food Science University of Otago Dunedin New Zealand; ^4^ Centre for Addiction Research Faculty of Medical and Health Sciences, The University of Auckland Auckland New Zealand

**Keywords:** caregivers, child health, costs and cost analysis, FASD, fetal alcohol spectrum disorder

## Abstract

**Introduction:**

Fetal Alcohol Spectrum Disorder (FASD) is a lifelong neurodisability caused by exposure to alcohol in utero. It can have a severe impact on the affected child as well as their families, yet the costs associated are unclear. This scoping review sought to identify the costs associated with raising a child with FASD.

**Methods:**

A database search was conducted in July 2024 on PubMed, Scopus, Ovid Medline and Web of Science, searching for all empirical research on the “cost” to “caregivers” of raising a child with “FASD”. Articles were excluded if they did not outline the costs of FASD, or the effects of prenatal alcohol exposure, or if they did not contain parent/caregiver response. After the removal of duplicates, 421 unique articles were found based on the search criteria. Just three articles met the inclusion/exclusion criteria. Two additional publications were identified through citation checking. Thus, five articles were included in this review. Thematic analysis was used to interpret the findings and synthesise the results.

**Results:**

Personal costs ranged between USD$198.13‐CAD$6215,27 per person per year. These articles identified that parents incurred costs related to medical care, education, social services, productivity losses, externalising behaviours, other direct costs to the family, and psychosocial impacts on families. Differences were considered in relation to the child's age, age at the time of diagnosis, severity of disability, relationship to caregiver, location, and other demographic factors.

**Discussion and Conclusions:**

More research is needed to provide a more accurate estimate of the cost of raising a child with FASD.

## 1 Introduction

Fetal alcohol spectrum disorder (FASD) is an irreversible “lifelong disability caused by prenatal alcohol exposure” [1]. FASD is characterised by neurodevelopmental dysfunction typically affecting the person's motor skills, academic achievement, executive functioning, impulse control, emotional regulation, maladaptive behaviour and/or social communication skills [2].

People with FASD are at risk of poorer life outcomes than people without prenatal alcohol exposure [3]. They have higher rates of mental health concerns [3, 4], are overrepresented in the criminal justice system [5], have lower educational attainment [6, 7] and are more likely to be in receipt of welfare support [4]. The costs to both society and families are extensive but largely unknown. Although the costs are likely to be high, people with FASD contribute in positive ways to their families and society, revealing strengths and skills where they add value [8, 9].

Financial burden is described as the perception of strain or stress as a result of financial pressures [10]. These pressures may arise from direct care‐related expenses, associated/indirect costs such as loss of income, or intangible costs such as missed opportunities and impacts on mental health. Lee and Cagle [10] described a conceptual framework to capture the impact of financial burden. In their model, they describe five objective contributors to financial strain and stress (loss of job/income, loss of saving/assets, care‐related expenses, insurance and benefits, and budget constraints) and one objective component that counteracts these factors (resources). They proposed that one's coping mechanisms interact with these objective components to influence subjective perceptions of stress/distress, difficulty paying bills, concerns/worries and financial satisfaction. These same subjective concerns are common in caregivers of people with FASD where research has found a significant impact on caregiver quality of life [11, 12, 13, 14]. However, there is a gap in literature that quantifies the financial burden experienced by caregivers of people with FASD, particularly research that has investigated both the objective and subjective components of Lee and Cagle's framework of financial burden.

While little research has investigated the costs to caregivers, in 2012, Popova et al. [15] proposed a model for estimating the economic impact of raising a child with FASD. They proposed a model that considers the direct costs of healthcare, law enforcement, and other direct costs (e.g., education, welfare, respite, cost of diagnosis, and professional training and development). Their model included productivity losses and intangible costs (such as the impact of FASD on caregivers and other affected individuals). They argued that there is a need to measure these costs in terms of systems (health care, justice, education, social services, etc.) and life stages (e.g., infant, childhood, adulthood). While the model has not been utilised to identify costs incurred by families, they did trial this model in Canada by considering just three of the components—acute care hospitalisations, corrections costs, and costs of foster care—using administrative data and previous literature. They concluded that there are significant costs; however, their estimates were limited due to inaccurate prevalence/incidence rates and the considerable variability in costs based on life stage, severity of disability, and services utilised. This model proposes a comprehensive range of cost categories; however, it has only been applied to administrative data studies. It remains to be seen if this same model is relevant to assessing costs incurred by caregivers of people with FASD.

### 1.1 Societal Costs

Research investigating the costs of FASD is scarce, particularly in the Aotearoa New Zealand context. Internationally, societal costs have been the focus of previous reviews, where they have predominantly conducted analyses using pre‐existing official datasets such as government records and/or health records to determine estimates of costs incurred at a population level. Greenmyer et al. [16] conducted a systematic review and quantitative analysis of international literature to identify the cost to society for each person who had FASD. They found many studies that considered the societal costs had been conducted in Canada and the United States of America, with a few additional studies appearing from studies in Sweden and New Zealand. They argued that while the annual cost to society per person with FASD varied widely, from $2035 to $298,975 per year (local currencies of each country included in the review), the mean cost per person was calculated to be $23,804. Despite these figures emphasising the costs incurred by society and the significant variation between countries, they are believed to be a huge underestimate of the true costs.

In 2016, Easton et al. [17] conducted a study in New Zealand to estimate the costs to society by quantifying productivity losses incurred due to FASD‐attributable morbidity and premature mortality. They estimated losses to the country were between NZD$49 million to NZD$200 million annually. Further, a recent report, published by the New Zealand Institute of Economic Research (inc.), estimated this societal cost to be much greater, with around NZD$4.8 billion attributable to FASD alone in 2023 in New Zealand [18]. They calculated that in 2023, the Disability Adjusted Life Years lost due to FASD was 75,526 years, or between NZD$3.15 and NZD$4.79 billion, with the value of lost productivity alone at over NZD$131 million, or NZD$1963 per person with FASD.

A further barrier to accurate cost calculations is that many cost studies utilise prevalence data in these cost estimates; however, prevalence rates in New Zealand are largely unknown. Researchers have estimated that prevalence rates are between 1.1% and 3.9% of the New Zealand population, with estimated rates as high as 6.3% for Māori [1]. Lange and colleagues [19] concluded that countries with a prevalence above 1% could be considered to have an elevated prevalence rate comparatively. This highlights that FASD may be a significant source of potential harm in New Zealand. Despite these relatively elevated rates, these are likely an underestimation due to the barriers to accessing a diagnostic assessment and the stigma associated with a diagnosis of FASD. This lack of prevalence data means that even the best cost estimate would grossly underestimate the true cost of FASD to society and would still fail to reflect the financial cost to caregivers, for which research appears to be scarce.

### 1.2 Costs to Caregivers

While official data records provide information on some aspects of the costs associated with FASD, these costs have not considered the direct and indirect costs to caregivers and families, further highlighting that these costs are an underestimation. It is vital to consider the costs incurred by, and the support needs of, families in context to reflect the political and social environments in which people live. For example, there are additional pressures on families who may be experiencing material hardship or other sources of disparity that are influenced by government systems.

A recent qualitative study of 56 caregivers [20] outlined a range of emotional, financial, and health impacts on caregivers and a lack of financial support to assist with their costs; however, there are significant gaps in the research on the cost of FASD [18]. There are no quantitative studies outlining the full cost to caregivers, or the burden on families; societal costs are outdated, and the studies have largely been conducted abroad. Additionally, estimates have been based on prevalence rates, which are largely unknown. They further highlighted a need for research in this area.

### 1.3 Rationale

As a study of this nature has not been conducted previously, it is first important to identify all cost factors associated with raising a child with FASD to ensure that the study design considers all possible determining factors.

This scoping review aims to consider the past literature in related fields to inform future research. Thus, this scoping review sets out to answer the following research questions:

1. What costs are incurred by caregivers when raising someone with FASD?
2. Why is it necessary to measure the determining factors of cost?
3. How have studies measured the costs incurred by parents of people with FASD?
4. How would research on caregiver costs contribute to this field of research?

This scoping review aims to identify components of costs incurred by families raising a child with FASD and will include, but is not limited to, direct and indirect financial costs reported by parents and caregivers. This review focuses on costs incurred by parents and caregivers of people with FASD and, therefore, excludes societal costs or costs calculated using official databases, aiming to inform future research implemented with families themselves.

## 2 Methods

### 2.1 Protocol and Registration of Review

We conducted a comprehensive literature search to find publications investigating the caring and financialcosts incurred by parents, from the perspective of parents and/or caregivers. While this scoping review was not pre‐registered, the PRISMA‐ScR (PRISMA extension for Scoping Reviews) [21] guidance was followed to ensure appropriate procedures were followed (see Appendix A—PRISMA‐ScR Checklist).

### 2.2 Eligibility Criteria

After an initial search for keywords related to FASD, search terms were refined to maximise the identification of FASD‐related articles. The review was limited to empirical research published since January 2005, including peer‐reviewed articles and dissertations. This time frame was chosen as the use of the term FASD became more consistent and the criteria were clarified in the literature in 2005. Following an international review of the 1996 guidelines, recommendations for the standardisation of the term FASD [22] were published in January 2005. This was followed by the publication of Canadian practice guidelines later in 2005 [23]. This acknowledges the increased reliability in the definition of FASD used in publications since this date.

No publications were excluded based on language, location, quality or status. Database searches were conducted in PubMed, Ovid Medline, Web of Science, and Scopus, with the first search conducted on 10 May 2024 and the final search on 12 July 2024. Table 1 presents the search strategy for PubMed (see Table S1, Supporting Information, for a list of search strategies for each database included in this review).

**TABLE 1:** Example of the search strategy used in this scoping review.

Database	Search strategy
Pubmed	(“parent*”[Title/Abstract] OR “caregiver*”[Title/Abstract] OR “guardian*”[Title/Abstract] OR “child*”[Title/Abstract]) AND (“foetal alcohol”[Title/Abstract] OR “Foetal alcohol spectrum disorder”[Title/Abstract] OR “foetal alcohol syndrome”[Title/Abstract] OR “foetal alcohol effects”[Title/Abstract] OR “Fetal alcohol spectrum disorder”[Title/Abstract] OR “fetal alcohol syndrome”[Title/Abstract] OR “fetal alcohol effects”[Title/Abstract] OR “alcohol related neurodevelopment*”[Title/Abstract] OR “alcohol‐related birth defects”[Title/Abstract] OR “FAS”[Title/Abstract] OR “FASD”[Title/Abstract] OR “ND‐PAE”[Title/Abstract] OR “alcohol related neurodevelopmental disorder”[Title/Abstract] OR “ARND”[Title/Abstract]) AND (“cost”[Title/Abstract] OR “health economics”[Title/Abstract] OR “quality of life”[Title/Abstract] OR “economic*”[Title/Abstract] OR “expens*”[Title/Abstract] OR “finance*”[Title/Abstract]) AND 2005/01/01:3000/12/12[Date‐Publication]

### 2.3 Selection of Sources of Evidence

The database results were uploaded to Rayyan.AI software [24], where duplicates were removed, and then the first author completed the title and abstract screening. Articles were excluded if they did not focus on FASD or the effects of prenatal alcohol exposure, or did not outline the costs of FASD (e.g., they were focused on clinical care, prevention, pre‐pregnancy planning, miscarriages, screening or diagnosis of FASD). All empirical studies were eligible for inclusion; thus, review articles were excluded. Any articles deemed relevant to the review, or the content was unclear, based on the title and abstract screening, were obtained for full‐text review.

The full‐text review was then completed. In addition to the aforementioned criteria, articles were reviewed to ensure they utilised parent‐ and/or caregiver‐reported data and focused on personal costs; see Table 2 for the full inclusion/exclusion criteria. Personal financial costs were those costs incurred by parents or caregivers in relation to the care of the child with FASD (including loss of earnings, healthcare, parking and transport), as opposed to the costs incurred by government such as hospital subsidies, provision of benefits, special education, and institutional care. Articles that were included based on their discussion of the psychosocial impacts investigated the relationship between financial costs and the burden or strain on caregivers. These articles were selected based on the associated impact of financial costs. If financial costs or burdens were not discussed, then the articles were excluded from the review. The research team discussed the inclusion of any publication that remained in dispute and agreed on whether to include or exclude the publication from the review.

**TABLE 2:** Scoping review inclusion and exclusion criteria.

Inclusion criteria	Exclusion criteria
Title and abstract screening	Title and abstract screening
Published from 1 January 2005 and 12 July 2024	Published before 2005
Focus on FASD, or related terms	Not focused on FASD or the effects of prenatal alcohol exposure
Empirical research studies	Not outlining costs of FASD or prenatal alcohol exposure
Full text available	Clinician report or opinion
Full‐text review Parent/caregiver reported perspectives on financial cost and/or psychosocial impacts (where there is an explicit relationship to financial costs discussed).	Full‐text review Self‐report by person with FASD only Clinician report only Uses administrative data only Reports societal costs only

Abbreviation: FASD, fetal alcohol spectrum disorder.

### 2.4 Data Charting Process

Data extraction was conducted to identify the objectives of the study, the focus, the methodology used, the age of the person with FASD, the sample size, the recruitment method, the tools utilised, and the main conclusions. Reflexive thematic analysis, drawing guidance from Braun and Clarke [25], was utilised to synthesise the findings across the included studies, particularly focusing on the cost components discussed in each study.

At the outset, data familiarisation ensured full immersion with the data. Articles were then reviewed and coded in accordance with the research questions and the PRISMA‐SR guidelines. Each article was coded and reviewed at least twice by the first author. NVivo Software was used to assist in coding and charting the articles identified for inclusion in the review [26, 27].

In line with Braun and Clarke's phases for thematic analysis [28], codes were generated through an iterative process, with at least two coding rounds for each article. Codes and themes were generated through iterative reflection on the key concepts and through repeated engagement with the included articles. Themes were refined and named, taking into consideration the related literature, and informed by theoretical frameworks related to the impacts of financial burden and economic stress [10, 15]. The themes, some explicitly extracted, some implicitly generated, are presented in table format for simplicity.

As this was a scoping review that identified a small number of articles, duplicate data extraction by multiple reviewers was not conducted, nor was a critical appraisal of the individual sources. It is acknowledged that, as the first author generated the themes, the findings represent one person's synthesis of the articles and reflect their engagement with the literature.

## 3 Results

### 3.1 Selection of Sources of Evidence

**FIGURE 1:**
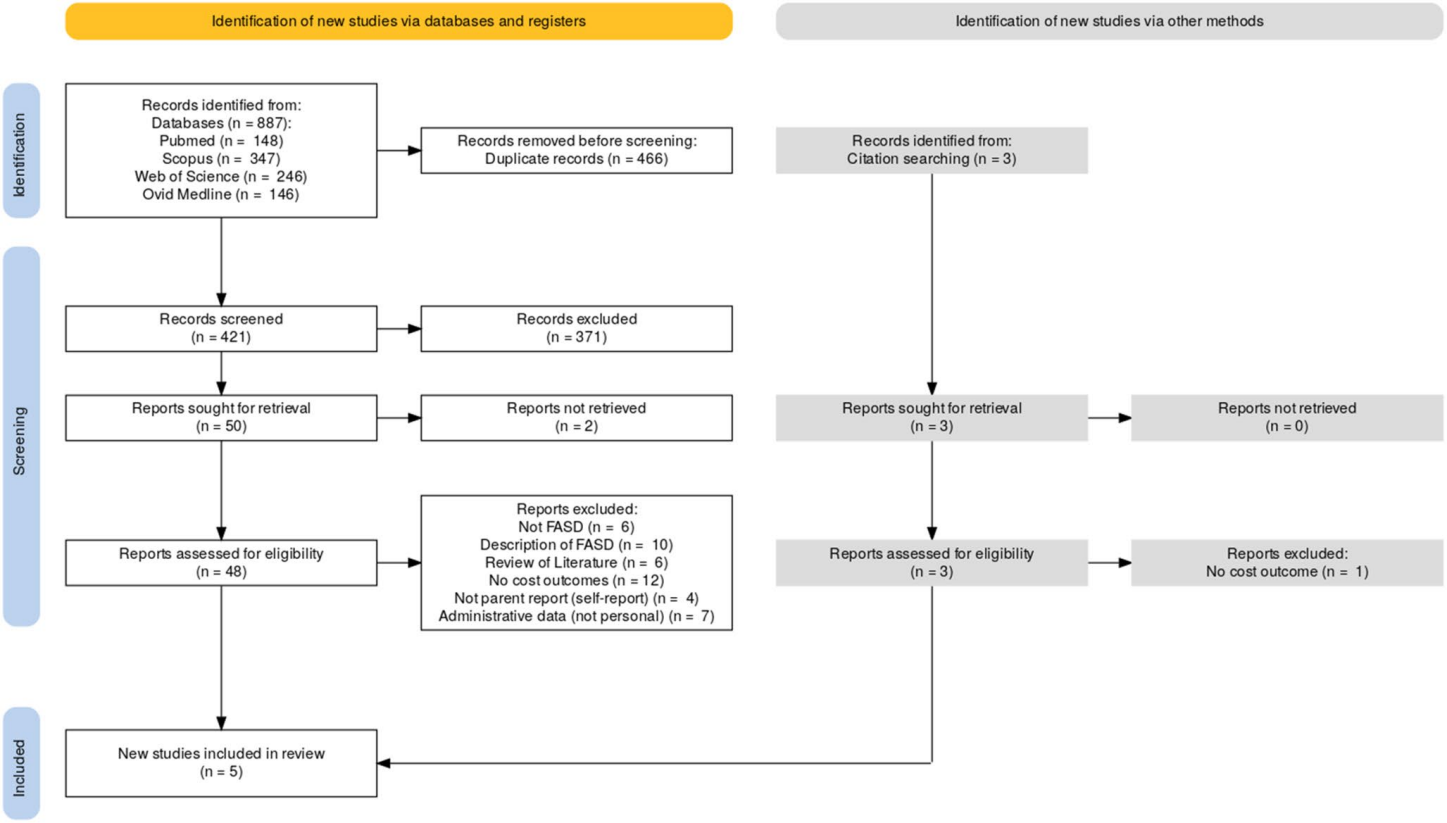
PRISMA Diagram representing the search strategy and identification of evidence included in the scoping review.

Through the database search, 887 articles were identified, and then references were uploaded to Rayyan.AI, which was the software used to facilitate the removal of duplicates (*n* = 466). Then, the titles and abstracts of 421 articles were screened; 374 publications were excluded in line with the aforementioned exclusion criteria. We could not obtain full‐text publications for two items; however, all remaining publications underwent full‐text screening (*n* = 48). The inclusion of three articles [11, 13, 14] was discussed within the research team, and all three were excluded as they focused on the psychosocial impacts of FASD with no explicit discussion of the costs associated with this. After a full‐text review, just three articles were deemed relevant to the research question by meeting the inclusion criteria. Further screening of the citation lists led to an additional two publications being identified, providing a total of five studies for inclusion in this review, see PRISMA diagram (Figure 1) for details [29].

### 3.2 Summary of Evidence

Five publications were identified for inclusion in the review; the characteristics of each study are summarised in Table 3.

**TABLE 3:** Characteristics of each source of evidence.

Sources of evidence	Objective of study	Focus	Location of study	Methodology	Age of person with FASD	Sample size	Recruitment method	Tools used	Cost component measures	Main findings (cost related)
Bobbitt et al. [33]	To evaluate caregiver stress and support needs	Access to services & barriers/Psychosocial impacts	Canada	Electronic questionnaire	No age restriction	125	FASD networks	Family Caregiver Survey Perceived Stress Scale	Medical Social services Productivity losses Psychosocial impacts on caregiver	71.2% of participants were at least moderately concerned about their financial situation. 70.2% were concerned about having to cover additional caring costs. Caregiver stress had a statistically significant correlation with caregiver financial situation.
Credé et al. [30]^a^	To identify and calculate the healthcare costs experienced by parents	Financial costs	South Africa	Medical record review and face‐to‐face survey interviews	< 12 years	44	Clinic databases	In‐house Questionnaire	Medical Education Social services Productivity losses Externalising behaviours Patient/family direct	USD$198.13 pp/year (2009). Medical costs were calculated as societal costs rather than personal expenses.
Hu and Da Silva [34]	To examine the oral healthcare services and barriers to access	Access to services & barriers	Canada	Electronic questionnaire	< 16 years	189	FASD networks	In‐house questionnaire based on access to care framework (Levasque)	Medical Social services Patient/family direct	63% of participants identified cost as a barrier to accessing dental care.
Stade et al. [31]	To estimate direct and indirect costs associated with FASD at the patient level.	Financial costs	Canada	Not explicitly stated—presumed survey interview	1–21 years	148	FASD networks	Health Services Utilisation Inventory	Medical Education Social services Productivity losses Externalising behaviours Patient/family direct	CAD$2454.46 pp/year (2003). Medical and education costs were calculated as societal/government expenses.
Stade et al. [32]	To calculate a revised estimate of direct and indirect costs associated with FASD at the patient level.	Financial costs	Canada	Not explicitly stated—presumed survey interview	< 53 years	240 caregivers (+10 adults with FASD)	FASD networks	Health Services Utilisation Inventory	Medical Education Social services Productivity losses Externalising behaviours Patient/family direct	CAD$4784.62 (2007) Plus $1430.65 in productivity losses. Additional medical, and education costs calculated for government funding

Abbreviation: FASD, fetal alcohol spectrum disorder.

^a^ This is a thesis, not a peer‐reviewed publication, and this study was conducted in a developing country (South Africa) and therefore the cost estimates may be different.

The main objective of three publications [30, 31, 32] was to calculate the related costs incurred by parents raising a child with FASD, the other two articles [33, 34] considered barriers to accessing healthcare and highlighted cost as one of the main barriers. Within each study, recall periods varied depending on the nature of the question, and most of the publications reported limiting the recall time to a ‘reliable period of recall’ [30, 31, 32].

The majority of the publications came from peer‐reviewed journals and recruited participants through FASD networks, except one publication [30]. The publication by Crede et al. [30] was a dissertation that recruited participants through a review of a clinic database. While the participants in all studies were parents and/or caregivers, each study differed by age range of the child with FASD, sample sizes, and the tools utilised to measure costs.

It was noted that two of the included studies [31, 32] were written by the same author, and the methodology was replicated. The authors argued that the 2009 publication was an improved estimate of the cost of raising a child with FASD as it included a greater age range. However, they stated that the study data utilised in each publication was unique and not replicated; therefore, both studies were included in this review.

It was noted that there was a significant variation in personal costs identified across the studies where this was the objective [30, 31, 32]. Personal costs varied from $198.13 [30] to $6215.27 [32] per person per year. The study with the lowest estimate was conducted in South Africa and has been discounted from previous economic calculations comparing the cost of FASD as it is a developing country with a vastly different economic status [16]. However, as this review focuses on cost experiences, the article was included in this scoping review. Each study calculated societal costs alongside personal costs, and the distribution between these two varied significantly. This suggests that the calculated costs reflected variations in the cost components included in each study as well as the social systems where the studies took place.

### 3.3 Synthesis of Results

Thematic analysis [28] was used to synthesise the cost components across the included studies.

#### 3.3.1 Cost Components

Any phrases within the articles that discussed financial costs or financial strain that lead to caregiver burden were coded. Codes were then used to generate themes. Table 4 summarises the findings and demonstrates that the main cost components identified were costs associated with medical care, education, social services, productivity losses, externalising behaviours, and direct costs to the family. Two articles did not capture costs incurred. Instead, they discussed the financial strain that leads to caregiver burden. These codes lead to the generation of the theme—psychosocial impacts on the caregiver.

**TABLE 4:** Cost components highlighted by each publication.

Cost component (Themes)	Type (Sub‐themes)	Reference
Medical	Barriers to accessing healthcare	[31, 32, 33, 34]
	Frequency of use of healthcare	[30, 34]
	Medical costs	[30, 31, 32, 33, 34]
	Type of healthcare facilities	[30, 32, 34]
Education	Home schooling, special schooling and/or tutoring	[30, 31, 32]
Social services	Residential care (number of care arrangements and/or type)	[31, 32]
	Unmet support service needs	[30, 33, 34]
Productivity losses	Days off work to care for child and attend appointments	[30, 31, 32, 34]
Externalising behaviours	Costs related to antisocial behaviour	[30, 31, 32]
Direct costs to family	Transportation and parking costs	[30, 31, 32, 34]
Psychosocial impacts on caregiver	Physical and emotional well‐being of the caregiver, stress levels, symptoms of anxiety and depression, sleep disturbances, financial stress, impact on relationships, social isolation	[33, 34]

Medical costs were a large focus of the studies identified in this review, with all five publications focusing on medical expenses. These costs related to attending appointments, accessing diagnostic tests, medication fees, medical devices, therapy fees, dental treatment, and hospital admissions. Many of the studies also included questions about the frequency [30, 34] and type of healthcare [30, 32, 34] to assist in calculating the costs of attending these appointments. Four publications [31, 32, 33, 34] also asked about barriers to accessing healthcare, highlighting a combination of costs, attitudes, stigma, and behaviour as some of the barriers to accessing medical services. Social service costs, including residential care (respite care, foster care and adoption costs) [31, 32] and unmet support service needs [30, 33, 34] were also a common theme.

Productivity losses and Other direct costs were a major focus, with four of the studies investigating this [30, 31, 32, 34]. These are the costs incurred due to caregivers taking days off work to care for the child with FASD, including the travel time, waiting time, duration of appointments and the child's number of sick days, as well as the other time spent caring for the child. These losses accounted for the greatest percentage of costs in all three studies that aimed to provide a cost estimate [30, 31, 32]. Other direct costs represented the costs incurred by families to attend appointments and care for their child that are not directly associated with any one activity, such as transportation and parking costs.

Three studies considered costs related to Education [30, 31, 32], such as homeschooling, special schooling, or accessing tutoring. These studies also identified Externalising behaviours [30, 31, 32] as a significant cost component; this includes costs related to the child engaging in anti‐social behaviour, such as legal aid and damage to property, stealing, and acts of aggression.

Just two of the included articles [33, 34] considered caregiver burden in relation to financial strain. These studies highlighted the significant psychosocial impacts on caregivers as a result of raising a child with FASD. Neither of these studies aimed to calculate the costs incurred but instead discussed the impact of financial pressures. Most parents in the study by Bobbit et al. [33] were worried about covering the extra costs required to care for their child with FASD. They reported impacts on the interpersonal relationships of caregivers, sleep, and perceived stress. Similarly, Hu and Da Silva [34] identified caregiver stress and financial strain as barriers to healthcare. This aligns with other studies which have found that raising a child with FASD impacts caregiver and family quality of life [11, 13, 14].

No consistency was evident across the studies with regards to the tools utilised to collect financial cost data, as each study utilised different tools to measure the financial costs. The two studies by Stade and colleagues [31, 32] both utilised the Health Services Utilisation Inventory [35], while Crede and colleagues [30] developed a questionnaire in‐house to determine costs.

Considerable variety in the tools was also evident in the assessment of caregiver burden and psychosocial impacts on caregiver health. Bobbitt et al. [33] utilised the Family Caregiver Survey [36] as cited by Bobbitt et al. [33] and the Perceived Stress Scale [37], while Hu and Da Silva [34] developed their own questionnaire, based on Levesque's Access‐to‐care Framework [38]. The inconsistency across these studies with regards to the choice of tool use demonstrates that there are a variety of tools available, and no ‘gold standard’ in this field of research has been established. Instead, it may be more appropriate to ensure that all the relevant cost components are considered and included, rather than focus on an individual tool.

#### 3.3.2 Demographic and Background Information

In addition to the key cost indicators, each study collected child and caregiver demographics, which could be considered during the analysis. As seen in Table 5 (parent/caregiver demographics) and Table 6 (child demographics), each study asked participants for background information to identify factors influencing costs incurred.

**TABLE 5:** Parent/caregiver demographics and background information.

Category	Category	Reference
Identity and background	Age	[30, 31, 32]
	Ethnicity	[31, 32]
	Gender	[30, 31, 32, 33]
	Home language	[30]
	Parental education	[30, 31, 32, 33, 34]
Home life	Household composition (number of people with FASD)	[33]
	Location/rurality	[31, 32, 33, 34]
	Marital status	[31, 32, 33]
	Relationship to child	[30, 31, 32, 33, 34]
Finances and employment	Employment status	[30, 32]
	Household income	[30, 32, 33, 34]
	Individual income	[30, 31, 32, 33]
	Medical insurance	[30, 34]
	Occupation	[31, 32]
	Grants/benefits	[30]

Abbreviation: FASD, fetal alcohol spectrum disorder.

**TABLE 6:** Child demographics and background information.

	Category	Reference
Identity	Age	[30, 31, 32, 33, 34]
	Ethnicity	[31, 32]
	Gender	[30, 31, 32, 33, 34]
	Home language	[30]
Home and school life	Length of time in the current home	[31, 32, 33]
	Relationship to parent/caregiver	[30, 31, 32, 33, 34]
	Residence status (living with caregiver)	[33]
	School type, grade and attendance	[30]
Health history	Age at the time of FASD diagnosis	[31, 32]
	Birth weight	[30]
	FASD diagnosis	[30, 31, 32, 33]
	Medical anomalies (e.g., heart defects)	[31]
	Medication use	[31, 32]
	Severity of disability	[31, 32]

*Note*: One study, not included in the review, concluded that the severity of disability may also include comorbid diagnoses and the number of traumatic events since birth [11].

Abbreviation: FASD, fetal alcohol spectrum disorder.

These background and demographic details varied between studies, but thematic analysis identified common themes. Identity, home and school life, caregiver finances and employment, and child's health history were consistent across studies; however, the depth of questioning varied. Each study shared summary characteristics of the participants based on these themes; however, none of the studies analysed these data in relation to costs with the aim of drawing conclusions. Studies discussed in their literature review or discussion that the age of the person with FASD [31, 32], the severity of disability [31, 32], the relationship of the caregiver [32], the geographical location [31] and whether the caregiver received a social support grant [30] may influence costs, but none of the studies analysed cost data in relation to these factors. They also did not discuss costs in relation to other factors such as gender, ethnicity, or experiences of material hardship, which are additional unexplored factors that may influence costs. This highlights a significant gap in the research, highlighting the importance of future research capturing a broad range of possible determining factors.

## 4 Discussion

### 4.1 Summary of Evidence

This scoping review identified five articles that discussed the personal financial costs for caregivers associated with raising a child with FASD. The limited evidence identified through this review concluded that caregivers of people with FASD incur significant costs across many areas of their lives. This research identified parental and caregiving costs related to health, education, social services, loss of productivity, externalising behaviours, and other direct costs to families. The magnitude of costs identified in these studies varied significantly but was limited by the lack of variability in the locations of the studies. This review supports the conceptual frameworks proposed by Lee and Cagle [10] and Popova et al. [15], both of which emphasise the need to capture broad cost components due to significant impacts of financial burden, spanning multiple domains.

This review demonstrated that financial burden impacts psychosocial well‐being, which may further perpetuate financial concerns. This finding is supported by other studies which identified links between raising a child with FASD and poorer outcomes related to caregiver well‐being and quality of life [11, 12, 13, 14]. The relationship between financial burden and psychosocial well‐being has not previously been quantified in relation to caring for someone with FASD. These studies pave the way for further investigation into the relationships between quality of life, mental well‐being, and the financial costs of raising a child with FASD. Additional costs, such as healthcare for the caregiver and productivity losses due to mental health concerns, may be exacerbated by the stressors of raising a child with FASD. These flow‐on effects should also be considered in future research.

Our understanding of the personal costs associated with raising a child with FASD is limited by the types of costs that have been considered in these studies. These studies focused primarily on healthcare costs, which are more readily quantifiable than costs associated with social service needs. In addition, qualitative studies such as Gibbs [20] highlight the significant financial costs of the unmet social services needs of families with FASD. These unmet needs represent a significant hidden cost to raising a child with FASD that may be difficult to quantify.

This review raises important implications for policy and practice. Policymakers need to recognise the far‐reaching impacts of financial burden and provide financial support to counterbalance the negative impacts on caregivers. Existing financial structures (such as the Child Disability Allowance [39] or the Disability Allowance in New Zealand [40]) should be reviewed to ensure they are easily accessible by caregivers of people with FASD. These financial structures may positively impact individuals while reducing burdens on other aspects of the healthcare and social service systems.

This review highlights the important role services have in providing support through accessible and affordable respite care, mental health support, and FASD caregiver support groups. Bobbitt et al. [33] found that 89% of caregivers would like more support in their role. If additional support were provided, there might not be the same financial burden and stress experienced by caregivers. Services play a key role in reducing the negative impacts of caregiving by ensuring caregivers are supported and enabled to live fulfilling lives.

The effects on caregivers may not be equal across demographic groups; however, these associations have not been investigated. Studies argued that various determining factors may impact the costs incurred by families. These included the child's age, the severity of disability, the relationship of the caregiver, geographical location, and whether the caregiver received a social support grant [30, 31, 32]; however, the explicit exploration of these data in relation to caregiver costs was missing.

This review found considerable variability in the cost conclusions. The inconsistencies across studies are likely related to methodological, temporal, and location differences. Variations existed across social and political contexts that reflected different social support systems, with the addition of within‐country differences evident through changes in healthcare systems and funding over time. This highlights the need for contemporary context‐specific research that acknowledges the local and national political environments at the time of the study.

Drawing on Lee and Cagle's conceptualisation of financial burden [10], future FASD research must consider the multiple domains that have the potential to impact financial burden. They emphasise that the trajectory of disease or illness, not visible in their model, also needs to be taken into account, and they argue that this can have a profound impact on the financial burden.

### 4.2 Limitations

A significant limitation of this study was that only the first author conducted the title and abstract screening. It is recognised that this may introduce bias; every effort was made to include all articles that discussed both costs and FASD. All articles for which inclusion was unclear were brought to the last author for discussion, and consensus was reached.

Another limitation of this scoping review was the limited sources of evidence identified in this field of research. This small number of identified studies included in this review was further limited by the lack of diversity in the context of the studies, with four out of the five studies conducted in Canada [31, 32, 33, 34], and the age of the studies, most of which were conducted before 2012. The limited scope was not surprising but does limit the validity and generalisability of the findings. As these studies were conducted predominantly in Canada and before 2012, the magnitude of these costs at the current time and the costs incurred in other contexts remain unknown.

Methodological rigour was not assessed in this scoping review. It was noted that two of the studies [31, 32] did not sufficiently describe their methodology, so it is unclear how these data were collected, bringing findings into question. As this scoping review aimed to identify the cost components for future studies rather than discuss the implications of the results, these issues of methodological rigour did not prevent their inclusion in the study. They are, however, important considerations for the application and understanding of the cost implications identified.

The identified studies focus on calculating the average costs to all families. However, there are significant gaps in these cost calculations. Differences between sociodemographic groups, such as ethnicity or household deprivation, were not factored into these cost calculations. They also do not take into account the hidden costs such as loss of opportunities, mental health concerns, experiences of trauma, and feelings of shame or embarrassment [20]. These factors require careful consideration and should be included in future research studies. The limitations of this scoping review only further highlight the knowledge gap that needs to be addressed.

## 5 Conclusion

This scoping review, the first of its kind, identified a gap in the literature that considers the financial and caring costs incurred by caregivers associated with raising a person with FASD. While the existing evidence recognises that there are significant costs in raising a child with FASD, the actual costs incurred by caregivers have not been clearly identified. We argue that without an estimate of parental costs, the impact of FASD will not be fully understood. With growing appreciation for the many areas of life impacted by raising a child with FASD, it is important that future research also reflects these diverse cost considerations. Gathering data on the caregiver and child's background, identity, home and school life, caregiver finances and employment, and the child's health history is essential.

This scoping review has highlighted a significant gap in research and identified areas for future study. It opens the door for future research, which should seek to find out the financial costs to caregivers alongside the psychosocial impacts that have a financial implication.

## Author Contributions

**Josie Tait:** conceptualisation, methodology, formal analysis, data curation, writing – original draft, writing – review and editing. **Anita Gibbs:** conceptualisation, methodology, writing – original draft, writing – review and editing. **Jessica McCormack:** conceptualisation, writing – original draft, writing – review and editing. **Holly Wilson:** conceptualisation, writing – original draft, writing – review and editing. **Joanna Ting Wai Chu:** conceptualisation, methodology, formal analysis, supervision, writing‐original draft, writing – review and editing.

## Conflicts of Interest

The authors declare no conflicts of interest.

## Supporting information


**Table S1.** Search strategy for scoping review.

**Appendix A.** PRISMA‐ScR Checklist.

## Data Availability

Data sharing is not applicable to this article as no new data were created or analyzed in this study.
