# Supplementary material for: Fetal Alcohol Spectrum Disorder: The Caring and Financial Burden to Caregivers—A Scoping Review
Source: Drug Alcohol Rev. 2025 May 4;44(5):1468–79. doi: 10.1111/dar.14071 (PMC12228032; doi:10.1111/dar.14071)
Supplement: Supplementary file 1 — Table S1. Search strategy for scoping review. [file DAR-44-1468-s002.docx]

## Table S1. Search strategy for scoping review

| Database | Search strategy |
| --- | --- |
| Pubmed | (("parent*"[Title/Abstract] OR "caregiver*"[Title/Abstract] OR "guardian*"[Title/Abstract] OR "child*"[Title/Abstract]) AND ("foetal alcohol"[Title/Abstract] OR "Foetal alcohol spectrum disorder"[Title/Abstract] OR "foetal alcohol syndrome"[Title/Abstract] OR "foetal alcohol effects"[Title/Abstract] OR "Fetal alcohol spectrum disorder"[Title/Abstract] OR "fetal alcohol syndrome"[Title/Abstract] OR "fetal alcohol effects"[Title/Abstract] OR "alcohol related neurodevelopment*"[Title/Abstract] OR "alcohol-related birth defects"[Title/Abstract] OR "FAS"[Title/Abstract] OR "FASD"[Title/Abstract] OR "ND-PAE"[Title/Abstract] OR "alcohol related neurodevelopmental disorder"[Title/Abstract] OR "ARND"[Title/Abstract]) AND ("cost"[Title/Abstract] OR "health economics"[Title/Abstract] OR "quality of life"[Title/Abstract] OR "economic*"[Title/Abstract] OR "expens*"[Title/Abstract] OR "finance*"[Title/Abstract]) AND 2005/01/01:3000/12/12[Date - Publication]) |
| Scopus | ( TITLE-ABS-KEY ( "foetal alcohol" OR "foetal alcohol spectrum disorder" OR "foetal alcohol syndrome" OR "foetal alcohol effects" OR "fetal alcohol spectrum disorder" OR "fetal alcohol syndrome" OR "fetal alcohol effects" OR "alcohol-related neurodevelopment*" OR "alcohol-related birth defects" OR "FAS" OR "FASD" OR "ND-PAE" OR "alcohol related neurodevelopmental disorder" OR "ARND" ) AND TITLE-ABS-KEY ( cost OR economic* OR finance* OR "quality of life" OR "expens*" OR "health economics" ) AND TITLE-ABS-KEY ( parent* OR caregiver* OR guardian* OR child* ) ) AND PUBYEAR > 2004 AND PUBYEAR < 2025 |
| Ovid Medline | ("foetal alcohol" or "foetal alcohol spectrum disorder" or "foetal alcohol syndrome" or "foetal alcohol effects" or "fetal alcohol spectrum disorder" or "fetal alcohol syndrome" or "fetal alcohol effects" or "alcohol-related neurodevelopment*" or "alcohol-related birth defects" or "FAS" or "FASD" or "ND-PAE" or "alcohol related neurodevelopmental disorder" or "ARND").ab. or ("foetal alcohol" or "foetal alcohol spectrum disorder" or "foetal alcohol syndrome" or "foetal alcohol effects" or "fetal alcohol spectrum disorder" or "fetal alcohol syndrome" or "fetal alcohol effects" or "alcohol-related neurodevelopment*" or "alcohol-related birth defects" or "FAS" or "FASD" or "ND-PAE" or "alcohol related neurodevelopmental disorder" or "ARND").kw. or ("foetal alcohol" or "foetal alcohol spectrum disorder" or "foetal alcohol syndrome" or "foetal alcohol effects" or "fetal alcohol spectrum disorder" or "fetal alcohol syndrome" or "fetal alcohol effects" or "alcohol-related neurodevelopment*" or "alcohol-related birth defects" or "FAS" or "FASD" or "ND-PAE" or "alcohol related neurodevelopmental disorder" or "ARND").ti.  AND  (cost or economic* or finance* or "quality of life" or "expens*" or "health economics").ab. or (cost or economic* or finance* or "quality of life" or "expens*" or "health economics").kw. or (cost or economic* or finance* or "quality of life" or "expens*" or "health economics").ti.  AND  (parent* or caregiver* or guardian* or child*).ab. or (parent* or caregiver* or guardian* or child*).kw. or (parent* or caregiver* or guardian* or child*).ti.  AND  2005-present |
| Web of Science | **parent* OR caregiver* OR guardian* OR child*** (Title) or **parent* OR caregiver* OR guardian* OR child*** (Abstract) or **parent* OR caregiver* OR guardian* OR child*** (Keyword Plus ®) or **parent* OR caregiver* OR guardian* OR child*** (Author Keywords)  AND  **cost OR economic* OR finance* OR "quality of life" OR "expens*" OR "health economics"** (Title) or **cost OR economic* OR finance* OR "quality of life" OR "expens*" OR "health economics"** (Abstract) or **cost OR economic* OR finance* OR "quality of life" OR "expens*" OR "health economics"** (Keyword Plus ®) or **cost OR economic* OR finance* OR "quality of life" OR "expens*" OR "health economics"** (Author Keywords)  AND  **"foetal alcohol" OR "foetal alcohol spectrum disorder" OR "foetal alcohol syndrome" OR "foetal alcohol effects" OR "fetal alcohol spectrum disorder" OR "fetal alcohol syndrome" OR "fetal alcohol effects" OR "alcohol-related neurodevelopment*" OR "alcohol-related birth defects" OR "FAS" OR "FASD" OR "ND-PAE" OR "alcohol related neurodevelopmental disorder" OR "ARND"** (Title) or **"foetal alcohol" OR "foetal alcohol spectrum disorder" OR "foetal alcohol syndrome" OR "foetal alcohol effects" OR "fetal alcohol spectrum disorder" OR "fetal alcohol syndrome" OR "fetal alcohol effects" OR "alcohol-related neurodevelopment*" OR "alcohol-related birth defects" OR "FAS" OR "FASD" OR "ND-PAE" OR "alcohol related neurodevelopmental disorder" OR "ARND"** (Abstract) or **"foetal alcohol" OR "foetal alcohol spectrum disorder" OR "foetal alcohol syndrome" OR "foetal alcohol effects" OR "fetal alcohol spectrum disorder" OR "fetal alcohol syndrome" OR "fetal alcohol effects" OR "alcohol-related neurodevelopment*" OR "alcohol-related birth defects" OR "FAS" OR "FASD" OR "ND-PAE" OR "alcohol related neurodevelopmental disorder" OR "ARND"** (Keyword Plus ®) or **"foetal alcohol" OR "foetal alcohol spectrum disorder" OR "foetal alcohol syndrome" OR "foetal alcohol effects" OR "fetal alcohol spectrum disorder" OR "fetal alcohol syndrome" OR "fetal alcohol effects" OR "alcohol-related neurodevelopment*" OR "alcohol-related birth defects" OR "FAS" OR "FASD" OR "ND-PAE" OR "alcohol related neurodevelopmental disorder" OR "ARND"** (Author Keywords)  Limit 2005-01-01 to 2025-01-01 |
